# Timing of High-glucose Diet in the *C. elegans* Lifecycle Impacts Fertility Phenotypes

**DOI:** 10.17912/micropub.biology.000614

**Published:** 2022-08-16

**Authors:** Amanda K. Engstrom, Caroline D. Davis, Jason L. Erichsen, Michelle A. Mondoux

**Affiliations:** 1 Department of Biology, College of the Holy Cross, Worcester, MA, USA

## Abstract

Human metabolic diseases and high-sugar diets have been associated with infertility. Previous studies show that high-glucose diet also affects fertility in *C. elegans,* leading to decreased offspring production and delayed reproductive timing. We tested whether the timing of glucose exposure affects these fertility defects or the embryo to larval transition. We found that decreased offspring production was strictly a response to high-glucose exposure in adulthood, whereas the delayed reproductive profile was influenced by both developmental and adult diets. We found no effect of high-glucose diet on the number of embryos that develop to the first larval stage. Together, these results suggest that the decreased offspring production and delayed reproductive profile may be separable phenotypes, and that a high-glucose diet reduces the number of offspring by interfering with processes regulated during adulthood.

**Figure 1. Decrease in live offspring on high-glucose diet is an adult phenotype that is partially separable from reproductive delay, and is not caused by defects in the larval transition .**
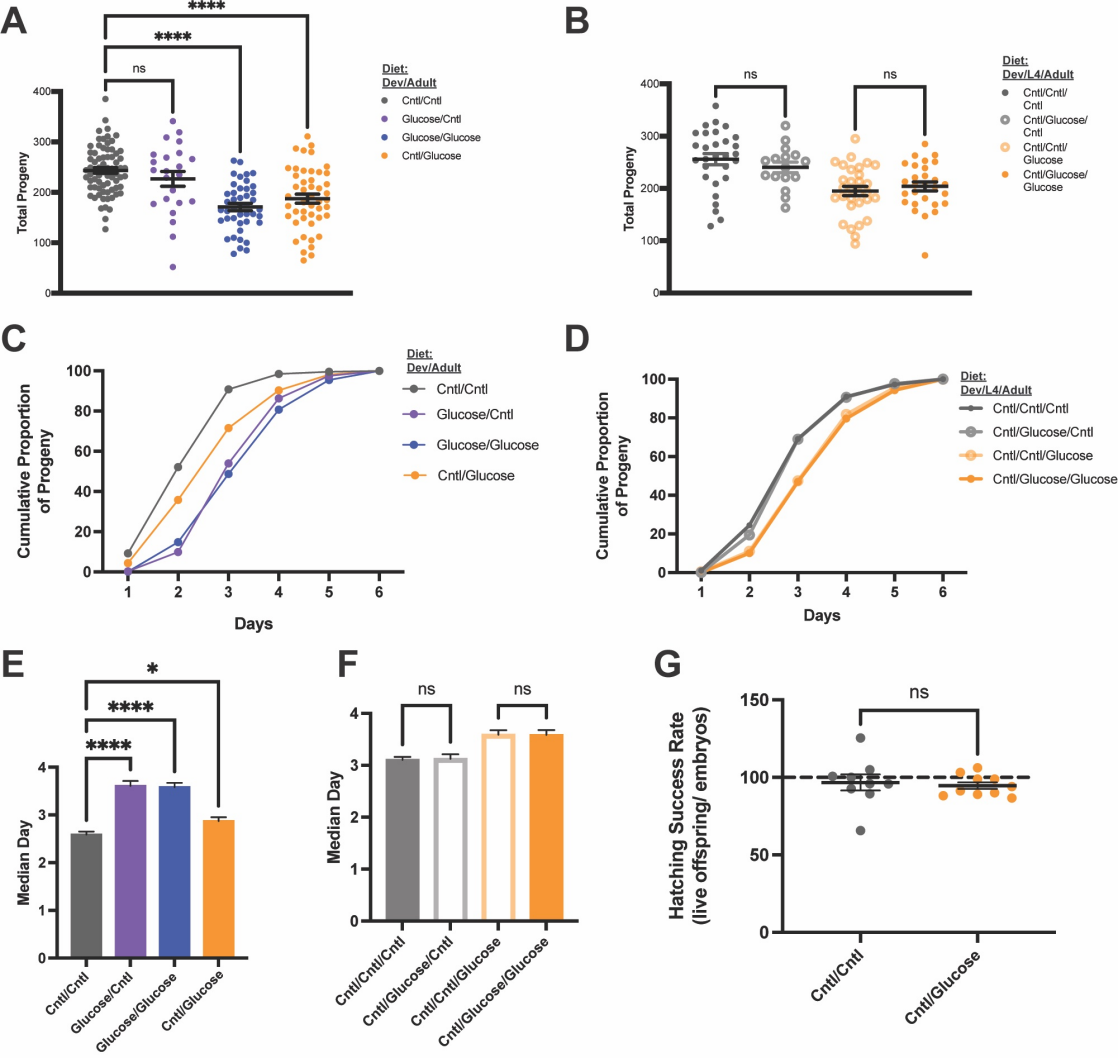
Wild-type (N2) *C. elegans* were exposed to a high-glucose diet at 20°C at different lifecycle stages. Figures are labeled with the dietary conditions in the given exposure time: embryo-L4/L4-adulthood (Dev/Adult; **A, C, E, G** ) and embryo-L4/L4-adulthood/adulthood (Dev/L4/Adult; **B, D, F** ). **(A) Exposure to high-glucose diet in adulthood reduces progeny production.** Animals on a high-glucose diet only during development (purple) produced the same number of offspring as those on a control diet (Cntl) in both development and adulthood (gray). Animals on a high-glucose diet during adulthood (blue and orange) had significantly fewer offspring, independent of developmental diet (****p < 0.0001). **(B) High-glucose diet in L4 alone does not affect progeny production.** Animals on a high-glucose diet only during the L4 stage (gray open points) produced the same number of offspring as those on a control diet throughout development and adulthood (filled gray points). Animals fed high-glucose diet in adulthood had decreased progeny production (as in A), whether they were exposed to control diet (open orange points) or high-glucose diet (filled orange points) in L4. **(C) High-glucose diet during development and adulthood both contribute to reproductive delay.** Proportion of total progeny produced daily from (A). Animals on a high-glucose diet during development (purple and blue) produced progeny more slowly, regardless of adulthood diet. Animals on high-glucose diet only during adulthood (orange) produced progeny more slowly than animals on a control diet throughout their lifecycle, but faster than animals on high-glucose diet during development. **(D) High-glucose diet in L4 alone does not alter reproductive timing.** Proportion of total progeny produced daily from (B). Animals fed high-glucose diet in adulthood had a reproductive delay (as in C), whether they were exposed to control diet (open orange points) or high-glucose diet (filled orange points) in L4. **(E) High-glucose diet during development and adulthood both shift the median reproduction point** . Day of adulthood at which each animal reached 50% of their total progeny production *.* Glucose exposure during development (purple and blue) resulted in a 1-day delay of the reproductive profile compared to control diet throughout the lifecycle (gray; ****p < 0.0001). Glucose in adulthood only (orange) delayed reproduction by 0.3 days (* p < 0.03). **(F) High-glucose diet in L4 alone does not alter the median reproduction point** *.* Glucose exposure during L4 alone (gray outline), had no effect on the reproductive profile compared to control diet throughout the lifecycle (solid gray). **(G) High-glucose diet does not affect the embryo to larval transition.** Embryos produced by animals on high-glucose diet in adulthood (orange) had equal hatching rates compared to embryos produced by animals on a control diet (gray).

## Description

High-sugar diets and metabolic disease are known to have negative effects on the reproductive system and have been associated with human infertility (reviewed in Sinclair and Watkins 2013). In *C. elegans,* a high-glucose diet has been shown to lead to fertility defects, including a decrease in the brood size, defined as the number of live offspring per adult, of self-fertilizing hermaphrodites (Lu and Goetsch 1993; Lee *et al* . 2009; Mondoux *et al.* 2011). In addition to a reduction in total offspring, when exposed to a high-glucose diet throughout the lifecycle, the peak of reproduction is delayed (Mondoux *et al* ., 2011). Fertility is a complex phenotype, and which facets of fertility are affected by high-glucose diet, and the contributing effects of developmental vs. adult glucose exposure on fertility, have not been elucidated.

Previous studies tested fertility after glucose feeding throughout the lifecycle. To determine when in the lifecycle glucose affects reproduction, we allowed self-fertilizing wild-type (N2) hermaphrodites to develop from embryos to their final developmental larval stage (L4) on one diet, and then switched them to a second diet starting as late L4s (Figs. 1A, C, and E). High-glucose diet during development had no effect on the total number of offspring produced, as animals fed a high-glucose diet in development but a control diet in adulthood produced the same number of progeny as animals on a control diet throughout their lifecycle (Fig. 1A). Furthermore, animals exposed to a high-glucose diet only during adulthood had a 25-30% reduction in the number of offspring produced, similar to the reduction observed when fed high-glucose diet throughout the entire lifecycle (as observed in Mondoux *et al.* 2011; Fig. 1A).

Hermaphrodite sperm production occurs during the L4 stage of development, and the L4 to adult transition includes important events that could influence fertility, including germline proliferation, meiotic entry, and sex determination (reviewed in Hubbard and Greenstein 2005). To test whether high-glucose diet exposure specifically in the L4 stage or during the L4 to adult transition affected fertility, animals were allowed to develop from embryos on a control diet and then switched to new diets at L4 and again at day one of adulthood (Figs. 1B, D, and F). Exposure to a high-glucose diet only in the L4 stage and during the L4 to adult transition had no effect on the number of offspring produced (Fig. 1B). These results indicate that the decreased progeny production in response to a high-glucose diet is due to processes that occur during adulthood, and exposure to high-glucose diet during development does not affect this facet of fertility. Interestingly, high-glucose diet has a similar effect on *C. elegans* hermaphrodite lifespan: exposure to high-glucose diet during adulthood decreases hermaphrodite lifespan, but developmental exposure to glucose has no effect (Lee *et al* ., 2009).

We also examined whether timing of high-glucose diet exposure affects the reproductive profile. Animals on a high-glucose diet throughout their entire lifecycle have a delayed reproductive profile, with the median point of the fertile period (when 50% of the offspring have been produced) occurring later (Figs. 1C and 1E and Mondoux *et al.* 2011). In contrast to the effects of high-glucose diet on reducing the total number of offspring, which is a phenotype resulting from exposure during adulthood (Figs. 1A-B), we find that high-glucose diet during development is sufficient to delay the reproductive profile as much as exposure during the entire lifecycle. High-glucose diet exposure only in development led to a 1-day delay in the reproductive profile (median at 3.6 days compared to 2.6 days for animals fed a control diet throughout the lifecycle; Figs. 1C and 1E), which was equal to the delay for animals exposed to glucose throughout their lifecycle (also median 3.6 days, Figs. 1C and 1E). High-glucose diet only in adulthood had a smaller but significant effect on reproductive timing (median 2.9 days; Figs. 1C and 1E), as these animals produced progeny more quickly than those exposed to glucose in development, but were still reproductively delayed compared to control diet. As we observed for total fertility, high-glucose diet only during L4 and the L4 to adult transition did not affect reproductive timing (Figs. 1D and 1F). These results suggest that both developmental and adult exposure to a high-glucose diet affect the reproductive profile, but that there is not an additive effect: high-glucose diet during adulthood does not further slow reproduction if the animal was exposed to high-glucose diet during development.

*C. elegans* embryos begin to develop in the hermaphrodite uterus and are laid during gastrulation. Development continues *ex utero* within the protective eggshell, and the animal completes organogenesis and morphogenesis by the time of hatching into the first larval (L1) stage. To test whether high-glucose diet reduces the number of live offspring by interfering with embryonic development or larval transition, we assayed hatching by comparing the number of embryos laid to the number of live offspring produced. We found no difference in hatching rates on a high-glucose diet compared to control diet: the number of viable larval-stage animals relative to the number of embryos laid was ~100% on both diets (Fig.1G). This suggests that a high-glucose diet does not reduce fertility by disrupting embryonic development.

Taken together, our results indicate that the effects of a high-glucose diet on fertility are dependent on when in the lifecycle exposure takes place. The reduction in number of offspring is dependent on exposure to high-glucose diet during adulthood, and this effect on fertility is partially separable from the effects of a high-glucose diet on the reproductive profile, which is influenced by both developmental and adult exposure. Further, our results suggest that the total number of offspring produced by self-fertilization of *C. elegans* hermaphrodites does not stem from defects in embryonic development, embryo to larval transition (hatching), sperm production (which occurs in L4), or or sex determination, germline proliferation, or meiotic entry (which all occur during the L4 to adult transition). Although germline proliferation and meiotic entry continue into adulthood, animals exposed to high-glucose diet during the L4-adult transition but not in adulthood have no reduction in offspring, suggesting that these processes are not the drivers of this phenotype. Rather, aspects of fertility that are regulated in adulthood, like oocyte quality or quantity, are more likely to be the factors disrupted by a high-glucose diet. For example, high-glucose diet could increase germline apoptosis, which would result in fewer oocytes and thus fewer offspring.

## Methods

Fertility Assays :

All worm stocks were maintained on NGM (nematode growth medium) plates with *E. coli* OP50. High-glucose diet plates were made by adding supplemental glucose to a final concentration of 333 mM after autoclaving as described (Mondoux et al. 2011). Synchronous embryos were produced by hypochlorite-NaOH treatment of gravid adult wild-type N2 hermaphrodites grown on NGM plates. Synchronous embryos were allowed to develop on NGM (control diet) or high-glucose diet plates at 20°C. At the L4 stage, animals were singled to individual plates of the indicated diet and then transferred to fresh plates daily for their entire fertile period. Progeny were counted daily on each plate for each day of adulthood. Total number of broods counted: Cntl/Cntl 72, Glucose/Cntl 23, Glucose/Glucose 43, Cntl/Glucose, 47 (Figs. A & C); Cntl/Cntl/Cntl 29; Cntl/Glucose/Cntl 16, Cntl/Cntl/Glucose 30, Cntl/Glucose/Glucose 28 (Figs. B &D). Statistical analysis was done using GraphPad Prism software and Tukey’s Multiple Comparisons Test (number of progeny) and one-way ANOVA with Sidak’s multiple comparisons test (reproductive timing).

Hatching Assay :

Ten worms per condition were prepared as described above for a fertility assay. On day 2 of adulthood, adult worms were removed from the plate and the number of embryos counted. Embryos were returned to 20°C and the number of live offspring was counted ~48 hours later to calculate percent hatching. Statistical analysis was done using GraphPad Prism software and an unpaired t test with Welch’s correction.

## Reagents

The wild-type N2 Bristol strain was provided by the Caenorhabditis Genetics Center (CGC), which is funded by the NIH Office of Research Infrastructure Programs (P40 OD010440).

## References

[R1] Hubbard EJ, Greenstein D (2005). Introduction to the germ line.. WormBook.

[R2] Lee SJ, Murphy CT, Kenyon C (2009). Glucose shortens the life span of C. elegans by downregulating DAF-16/FOXO activity and aquaporin gene expression.. Cell Metab.

[R3] Lu, N.C., K.M. Goetsch. Carbohydrate requirement of *Caenorhabditis elegans * and the final development of a chemically defined medium. Nematologica. 1993. 39:1:303-311.

[R4] Mondoux MA, Love DC, Ghosh SK, Fukushige T, Bond M, Weerasinghe GR, Hanover JA, Krause MW (2011). O-linked-N-acetylglucosamine cycling and insulin signaling are required for the glucose stress response in Caenorhabditis elegans.. Genetics.

[R5] Sinclair KD, Watkins AJ (2013). Parental diet, pregnancy outcomes and offspring health: metabolic determinants in developing oocytes and embryos.. Reprod Fertil Dev.

